# Bowing Out of Shaking Hands?

**DOI:** 10.3201/eid2801.AC2801

**Published:** 2022-01

**Authors:** Nkuchia M. M’ikanatha, Byron Breedlove

**Affiliations:** Pennsylvania Department of Health, Harrisburg, Pennsylvania, USA (N.M. M’ikanantha);; Centers for Disease Control and Prevention, Atlanta, Georgia, USA (B. Breedlove)

**Keywords:** art science connection, emerging infectious diseases, art and medicine, about the cover, public health, Lilly Martin Spencer, Shake Hands?, Bowing Out of Shaking Hands?, transmission, drug-resistant microbes, Campylobacter spp., Salmonella spp., bacteria, antimicrobial resistance

**Figure:**
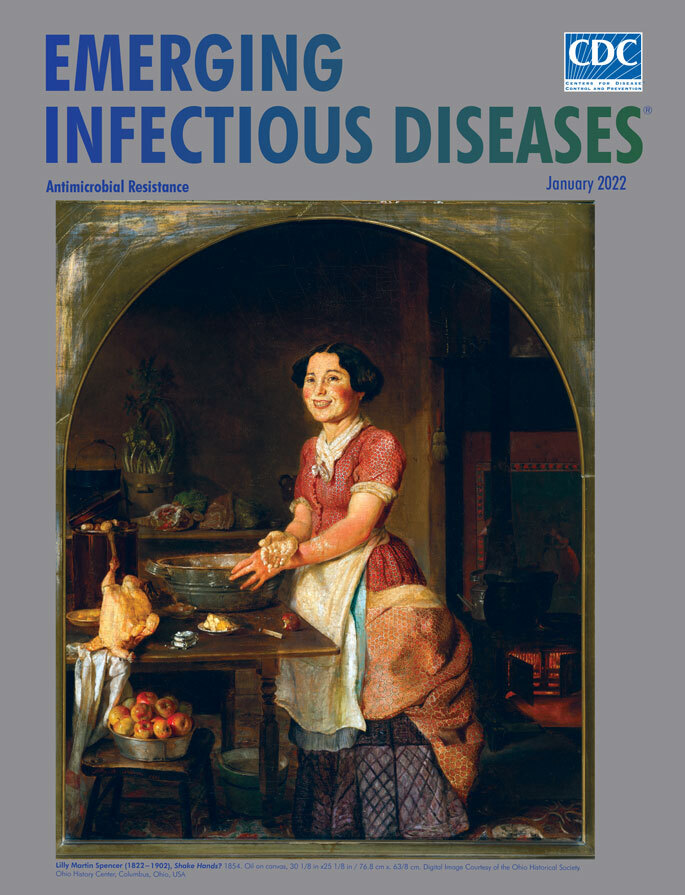
**Lilly Martin Spencer (1822−1902), *Shake Hands*? 1854.** Oil on canvas, 30 1/8 in x 25 1/8 in/76.8 cm x 63.8 cm. Digital image courtesy of the Ohio Historical Society, Ohio History Center, Columbus, Ohio, USA.

This month’s cover features *Shake Hands?* by Lilly Martin Spencer, one of the most recognized American artists of the mid-nineteenth century. In this painting, Spencer seems to be asking the viewer to ponder whether shaking hands is appropriate, a question that is currently pertinent considering the persistent and growing danger of antimicrobial resistance, which threatens to put us back into Spencer’s time when effective antimicrobials were not an option. 

Born in Exeter, England, in 1822 to French parents, Angélique Marie Martin was called by her nickname, Lilly. When Spencer (then Martin) was 8 years old, she immigrated with her parents to the United States, where they settled in the small town of Marietta, Ohio. Her progressive parents homeschooled her and encouraged her interest in art. Spencer demonstrated her talent at a young age by making charcoal drawings of family scenes. She later studied portrait painting under itinerant artist John Insco Williams, though she was largely self-taught. At age 22, she married Benjamin Rush Spencer. As the sole breadwinner, Spencer painted to support her family while her husband took care of domestic responsibilities, an uncommon arrangement in those days. 

Spencer’s sentimental art includes numerous scenes that offer the observer a glimpse of family life; she sometimes used herself, her husband, and her children as models. Considered one of Spencer’s signature pieces, *Shake Hands?* shows a smiling, neatly dressed cook extending her right hand, with its flour-coated palm up, and her left hand resting on a grey pail. With details including the presence of raw meat, a blood-stained white apron, and a half-eaten apple, the painting evokes a visceral response mixed with a dry sense of humor. Art historian Wendy J. Katz interprets the implicit question as “a test whether the viewer acknowledges her as a lady, or equal.” Katz sees the painting as Spencer’s response to criticisms of Americans by some European writers, such as Frances Trollope, for the American practice of shaking hands. 

Spencer’s sentimental genre became popular in the United States and Europe in the mid-1800s, and she displayed her paintings at the Philadelphia Centennial Exhibition in 1876. Spencer remains an iconic artist of her generation, and her works are exhibited at various museums, including the Ohio History Museum (Columbus) and the National Museum of Women in the Arts (Washington, DC).

Although its egalitarian ethos seems more American than other forms of greeting, such as bowing or hat doffing, the custom of shaking hands has existed for centuries and quite likely evolved as a peaceful gesture between strangers and as a way to seal agreements. Former diplomat and writer Andy Scott notes that Homer described handshakes several times in *The Iliad* and *The Odyssey*, written during the eighth century bce. He continues, “[Perhaps] the earliest representation can be found on a ninth-century bce stone relief from Mesopotamia, in today’s northern Iraq, showing the Assyrian King Shalmaneser III shaking hands with a Babylonian ruler to seal their alliance.” In ancient Rome, the handshake symbolized friendship and loyalty; pairs of clasped hands appeared on coins.

In modern times, shaking hands continues to have an important cultural role as a greeting among friends and colleagues and an introduction between strangers. Depending on circumstances, this practice may also enable transmission of drug-resistant microbes with potentially serious consequences. In 1861, seven years after Spencer painted *Shake Hands?,* the Hungarian gynecologist Ignaz Semmelweis published his well-known investigations into the role of hand hygiene in preventing person-to-person transmission of infectious agents Although others, such as the American physician-poet Oliver Wendell Holmes Sr., had recognized the infectious nature of certain diseases, Semmelweis was meticulous in documenting an association between handwashing and positive clinical outcomes. “Few readers of today would care to wade through [Semmelweis’ work] …The array of figures assembled to prove his case is enormous, convincing, complete…” wrote medical historian Robert Wiese. William Jarvis opined in 1994 in the *Lancet* that “Semmelweis deduced that infection was also transmitted by living organisms; he then insisted on handwashing… [but] at that time few doctors believed…” 

In the twentieth century, with better understanding of infectious etiologies, investigators elucidated the role of handshaking in spreading disease. In a 1926 study published in *The Public Health Journal*, researchers Hill and Mathews wrote, “In a series of experiments we have shown that organisms, both non-pathogenic and pathogenic, are transferred from one hand to another in hand shaking―a fact of great importance in the study of the spread of infection.” Contaminated hands play a large role in spreading foodborne pathogens of animal origin, such as *Campylobacter* and *Salmonella*, which are increasingly resistant to antimicrobial agents. 

The spread of antimicrobial-resistant disease-causing microbes to large populations, particularly through food prepared outside the home, remains a persistent public health problem. “The unwary cook also can easily transfer microbes from raw meat to other foods being prepared or make other food-handling errors that can lead to illness in the consumer,” wrote Alexandra Levitt and colleagues in *Silent Victories: The History and Practice of Public Health in Twentieth-Century America*. 

Present-day sobering realities call for altered meanings to Spencer’s *Shake Hands?* In community and healthcare settings, pathogens are often spread by contact. Therefore, hand hygiene is necessary before and after certain activities such as food preparation and patient care. Hand hygiene enables us to maintain human connection without inadvertently contributing to the spread of pathogens. 
